# Elderly onset atypical Lemierre’s syndrome concurrent with a rheumatoid vasculitis sacral ulcer infection: a case report

**DOI:** 10.1186/s12879-023-08089-z

**Published:** 2023-03-08

**Authors:** Koji Mishima, Kazunobu Aoki, Yusuke Shirai, Hiroshi Aritomo, Maiko Iwasaka, Masakazu Katsura, Tomofumi Tatsutani, Hiroko Ikeuchi, Kensuke Oryoji, Shinichi Mizuki

**Affiliations:** 1 Center for Rheumatic Diseases, Matsuyama Red Cross Hospital, 1 Bunkyomachi, Ehime Prefecture 790-8524 Matsuyama City, Japan; 2 Department of Respiratory Medicine, Japanese Red Cross Ishinomaki Hospital, Miyagi Prefecture Ishinomaki City, Japan; 3 Department of Otorhinolaryngology, Matsuyama Red Cross Hospital, Matsuyama City, Ehime Prefecture Japan; 4 Division of Dermatology, Matsuyama Red Cross Hospital, Matsuyama City, Ehime Prefecture Japan; 5 Department of Thoracic Surgery, Matsuyama Red Cross Hospital, Matsuyama City, Ehime Prefecture Japan

**Keywords:** Atypical Lemierre’s syndrome, *Streptococcus anginosus*, Sacral ulcer infection, Rheumatoid arthritis, Case report

## Abstract

**Background:**

Typical Lemierre’s syndrome is usually secondary to an oropharyngeal infection. Recently, several cases following a primary infection site other than the oropharynx have been reported as atypical Lemierre’s syndrome; although, these primary lesions are limited to the head and neck. This is the first case potentially sequential to infectious foci outside the head and neck.

**Case presentation:**

We describe an atypical Lemierre’s syndrome in a 72-year-old woman with rheumatoid arthritis, which occurred during the treatment of *Streptococcus anginosus* bacteremia acquired from a sacral ulcer infection related to rheumatoid vasculitis. At first, the symptoms resolved after the initial administration of vancomycin for the bacteremia caused by methicillin-resistant *Staphylococcus aureus* and *Streptococcus anginosus* that entered via a sacral ulcer. On the 8th day, the patient developed a fever of 40 °C and unexpectedly required 10 L of oxygen due to rapid deterioration of oxygenation temporarily. Immediately contrast-enhanced computed tomography was performed to investigate systemic thrombosis including pulmonary embolism. Afterward, the newly formed thrombi at the right external jugular vein, bilateral internal jugular veins, and the right small saphenous vein were detected, and apixaban was started. On the 9th day, the patient again had an intermittent fever of 39.7 °C, and continuous *Streptococcus anginosus* bacteremia was revealed; subsequently, clindamycin was administered. On the 10th day, she developed a left hemothorax; consequently, apixaban was discontinued, and a thoracic drain was inserted. She repeatedly had an intermittent fever of 40.3 °C, and contrast-enhanced computed tomography detected an abscess formation at the left parotid gland, pterygoid muscle group, and masseter muscle. After Lemierre’s syndrome was diagnosed in combination with the abovementioned jugular vein thrombus, clindamycin was replaced with meropenem, and vancomycin was increased. Swelling of the lower part of the left ear became prominent with delay and peaked at approximately the 16th day. The subsequent treatment course was favorable, and she was discharged on the 41st day.

**Conclusion:**

Clinicians should consider Lemierre’s syndrome as the differential diagnosis of internal jugular vein thrombosis occurring during sepsis, even though an antibiotic is administered or a primary infection site is anything besides the oropharynx.

## Background

Lemierre’s syndrome is a rare disease that usually affects healthy adolescents or young adults and occurs in around 3 per million people yearly, with a 2% mortality rate [1, 2]. Three features characterize Lemierre’s syndrome: (1) an oropharyngeal infection, (2) systemic septic embolism from the thrombophlebitis of the internal jugular vein, and (3) the presence of *Fusobacterium necrophorum* as the causal organism [3]. However, it has recently been proposed that the causative organism being *F. necrophorum* is not mandatory, as the involvement of various facultative and obligate anaerobes has also been reported [4]. Furthermore, sporadic reports of atypical Lemierre’s syndrome involving head and neck infections, excluding those of the oropharyngeal system, such as dental abscess and orbital cellulitis, exist [5–8]. Meanwhile, these primary infection sites are limited to the head and neck. To the best of our knowledge, we are the first to report a case of atypical Lemierre’s syndrome caused by *Streptococcus anginosus* potentially sequential to a sacral ulcer distant from the head and neck as the primary site of infection.

## Case presentation

A 72-year-old woman with Stage IV, Class IV rheumatoid arthritis of 10 years was undergoing treatment with Abatacept (500-mg IV every 4 weeks), only to fail adequate disease activity control. Three months before her admission, the patient had been hospitalized with a sacral and left lower extremity ulcer infection due to rheumatoid vasculitis. While we detected *Escherichia coli*, *Raoultella ornithinolytica*, and *Stenotrophomonas maltophilia* in her left lower leg, *Streptococcus anginosus* and *Peptostreptococcus anaerobius* were detected in the sacral region. Hence, levofloxacin was selected based on antimicrobial susceptibility and administered for 1 month, with dosages adjusted according to renal function. Subsequently, the left leg ulcer and the sacral fistula also began to shrink gradually. Thereafter, she was admitted to the hospital because of hip pain, general malaise, and anorexia after a fall. On admission, she had a temperature of 39.0 °C, blood pressure of 83/68 mmHg, a pulse of 85/min, SpO_2_ of 98% (room air), an ulcer with purulent discharge in the median sacral region, and a chicken-egg-sized ulcer with black and yellow necrotic tissue on the medial side of the left lower leg. Furthermore, she had elevated C-reactive protein (CRP) (19.7 mg/dL) and procalcitonin (12.6 ng/mL) levels, suggesting worsening sacral and left lower extremity ulcers and concomitant infection. As a result, rheumatoid vasculitis treatment was intensified by adding betamethasone (2 mg/day) and meropenem (MEPM) (1.5 g/day) was initiated. On the 2nd day, methicillin-resistant *Staphylococcus aureus* (MRSA) was detected in a blood culture taken on admission, then MEPM was replaced with vancomycin (VCM, 0.75 g/day, after the initial 1-g dose adjusted based on creatinine value 1.04 mg/dL and body weight 41 kg for target trough value 10–15 µg/mL). Additionally, the same blood culture confirmed *S. anginosus* on the 5th day. Nevertheless, her fever resolved after the 2nd day, CRP decreased to 3.7 mg/dL, the course of the disease recovery was good, and treatment was continued, except that the dose of VCM was increased to 1 g/day due to a low trough value of 3.6 µg/mL upon returning to normal renal function. Subsequently, in the pus from the sacral ulcer collected on admission, MRSA, *Streptococcus agalactiae*, *S. anginosus*, *Finegoldia magna*, and *Corynebacterium jeikeium* were also detected, indicating that sacral ulcers were the entry of the bacteremia.

**Fig. 1:**
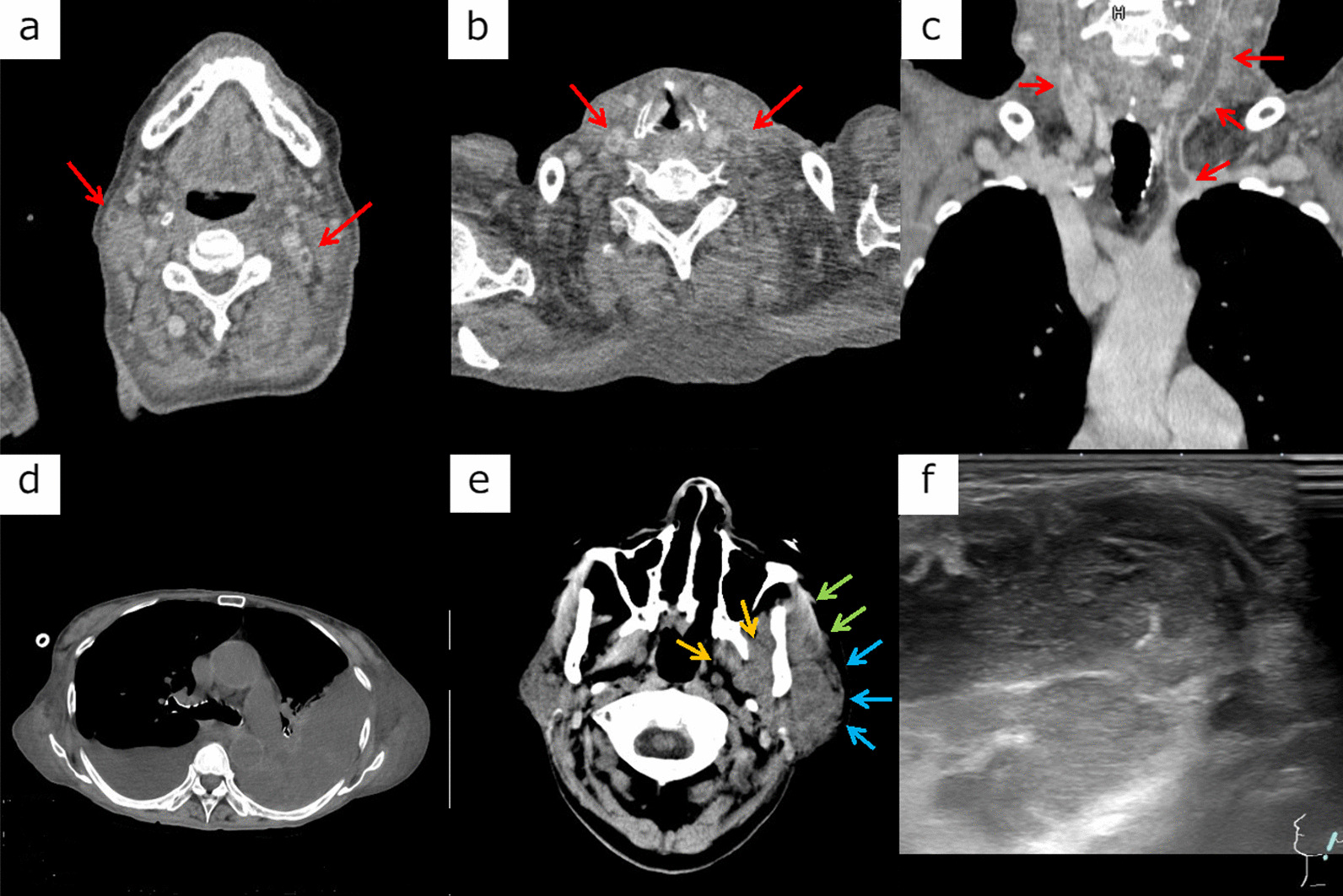
**a**–**c** On the 8th day, thrombi appeared in the right external jugular vein, bilateral internal jugular veins, and the right small saphenous vein. **d**, **e** On the 10th day, a left hemothorax appeared, and the left parotid gland (blue arrow), near the pterygoid muscle group (yellow arrow) and masseter muscle (green arrow), was enlarged. A slightly low internal absorption, suggesting abscess formation, was also observed. **f** Posterior echogenic enhancement with a well-defined border and heterogeneous internal echogenicity of about 37 × 35 mm near the left parotid gland, with findings showing an abscess after superficial ultrasound examination of the left parotid gland

On the 8th day, she developed a fever of 40 °C and suddenly required 10 L of oxygen due to rapid deterioration of oxygenation. Since D-dimer was elevated to 29.3 µg/mL, contrast-enhanced computed tomography (CECT) was immediately performed from neck to pelvis investigating systemic thrombosis including pulmonary embolism, revealing thrombi at the right external jugular vein, bilateral internal jugular veins, and right small saphenous vein without previously inserted a central venous catheter or clinical manifestations (Fig. 1a–c). Although, there was no evidence of pulmonary embolism. Therefore, apixaban (20 mg) was started according to the American Society of Hematology Venous Thromboembolism Guidelines. Although CRP was re-elevated to 7.2 mg/dL, her fever resolved within a few hours, and oxygen demand improved to SpO_2_ of 98% at around one liter of oxygen. Subsequently, only blood cultures including both aerobic and anaerobic were collected from the bilateral upper limbs, and the antimicrobial therapy regimen was unchanged.

**Fig. 2:**
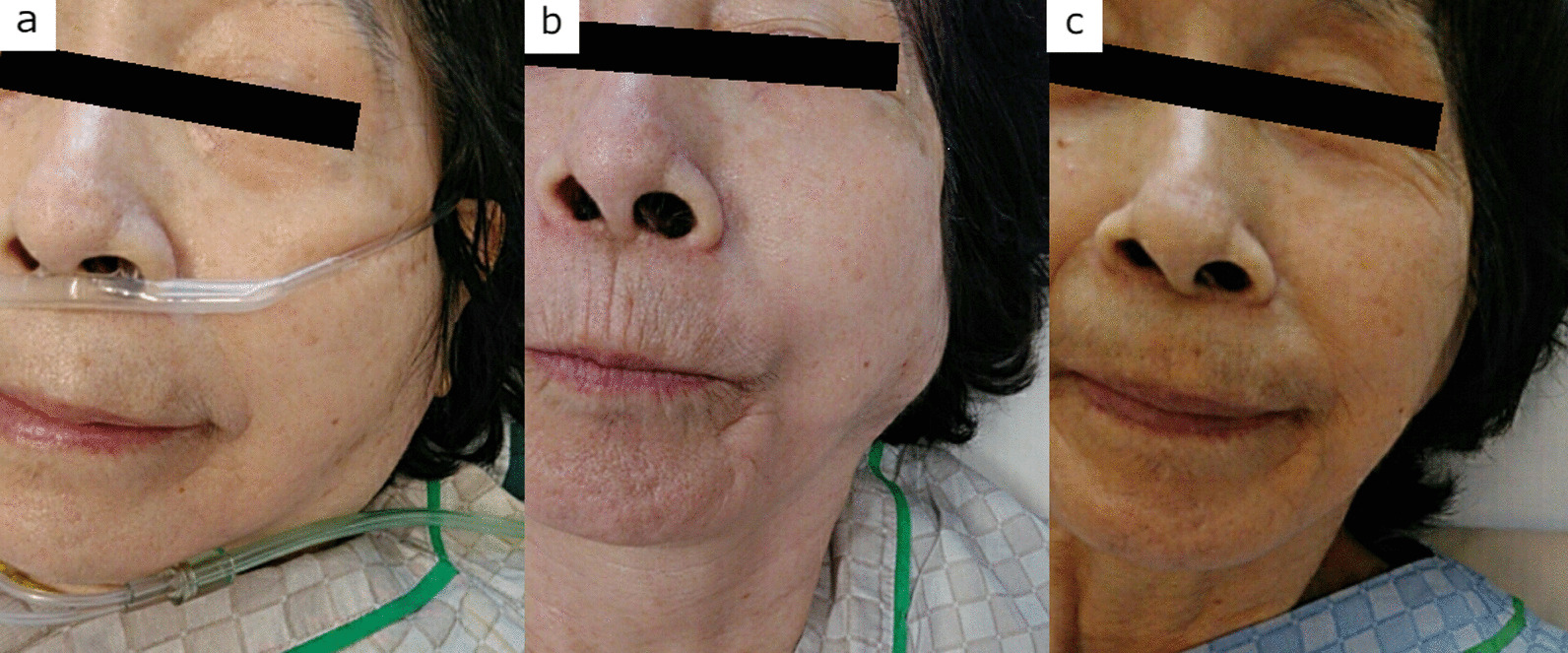
**a** On the 10th day, with the thrombus in the right external jugular vein and bilateral internal jugular veins, described 2 days before, the left parotid gland, pterygoid muscle group, and masseter muscle were enlarged, as shown by CECT. Induration of the left parotid gland was also observed. However, no swelling of the left parotid region on visual examination was detected. **b** On the 16th day, although swelling of the left parotid gland became prominent, no skin redness, inflammation of the buccal mucosa, or drainage of pus from the Stenon duct was observed. **c** On the 41st day, the swelling in the left parotid gland area disappeared

On the 9th day, however, clindamycin (CLDM) (600 mg/day) was initiated because she had an intermittent fever of 39.7 °C, and *S. anginosus* was continuously detected in the blood culture collected the day before. Then, on the 10th day, anemia progressed rapidly from hemoglobin 10.2 to 5.5 g/dL level, and a CECT scan revealed a left hemothorax (Fig. 1d), leading to discontinuation of apixaban and insertion of a thoracic drain. Moreover, in addition to an intermittent fever of 40.3 °C, worsening CRP elevation to 10.5 mg/dL was observed. Although swelling of the left parotid gland was not evident on a visual examination (Fig. 2a), CECT revealed an abscess formation at the left parotid gland, pterygoid muscle group, and masseter muscle (Fig. 1e). As a result, with the abovementioned jugular vein thrombus, Lemierre’s syndrome was diagnosed (right small saphenous vein lesion was considered a remote lesion of Lemierre’s syndrome or just a venous thrombosis due to hypercoagulation and immobilization), causing a change from CLDM to MEPM (3 g/day). VCM was also increased to 2 g/day because the trough value was 9.9 µg/mL, slightly lower than the target value. Then, transthoracic and transesophageal echocardiography was performed to exclude infective endocarditis, revealing no evidence of vegetation. Due to the diagnosis-based treatment modifications, the fever resolved after the 11th day, CRP decreased gradually, and oxygen administration was terminated on the 14th day. However, the lower part of the left ear became swollen with delay (Fig. 2b), and ultrasonography revealed the formation of an abscess (Fig. 1f). Thereafter, MEPM was de-escalated to CLDM (600 mg/day) on the 22nd day based on antimicrobial susceptibility. Moreover, although VCM and CLDM were terminated on the 30th day, 4 weeks after the MRSA blood culture became negative, amoxicillin (750 mg/day) for *S. anginosus* and minocycline (200 mg/day) for MRSA were initiated orally on the 31st day and continued for another 3 months due to infection under immunosuppressive therapy. An improvement at the left hemothorax was observed, and the thoracic drain was removed on the 27th day. Furthermore, the CRP became negative (0.15 mg/dL), the left lower ear mass disappeared (Fig. 2c), and the sacral and left lower leg ulcers tended to shrink. On the 41st day, she was finally transferred to continue rehabilitation.

## Discussion and conclusions

This case report presented a case of atypical Lemierre’s syndrome caused by *S. anginosus* during the treatment of *S. anginosus* and MRSA bacteremia potentially acquired from a sacral ulcer. It was atypical because in addition to age and species, the portal of entry might be anatomically distant from the head and neck. If that is the case, based on our extensive literature search, this is the first report of an atypical Lemierre’s syndrome with a portal of entry other than the head and neck region.

*Streptococcus anginosus* is a facultative anaerobic Gram-positive coccus that, along with *Streptococcus constellatus* and *Streptococcus intermedius*, constitutes the *S. anginosus group*. It is found in the upper respiratory tract, digestive system, and reproductive organs. Although *S. anginosus* is usually nonpathogenic, it can cause infections in compromised hosts and middle-aged or older adults with underlying diseases, such as solid tumors, type 2 diabetes, chronic kidney diseases, connective tissue diseases during immunosuppressive therapy, and central nervous system diseases (e.g., cerebrovascular diseases, traumatic brain injuries, myasthenia gravis, and Parkinson’s disease) [9]. Furthermore, *S. anginosus* often infects the hepatobiliary system, intra-abdominal cavity, chest, soft tissues, head, and neck [10]. Similar to *F. necrophorum*, *S. anginosus* forms invasive pyogenic lesions, possibly involving the following mechanisms. *S. anginosus* binds to fibronectin, a subendothelial matrix protein involved in cell–cell adhesion, which allows it to invade and settle in tissues [11]. It also attracts polynuclear leukocytes, resists phagocytosis and killing, and can survive post phagocytosis by polynuclear leukocytes, thereby forming an abscess [12]. Additionally, the surface molecules of *S. anginosus* promote platelet aggregation and fibrinogen binding forms platelet thrombi [13].

Had the original source of infection been the sacral ulcer, the mechanism that gave this patient atypical Lemierre’s syndrome is speculated to be as follows. Originally, considering the influence of gravity in blood flow, thrombosis is less likely to occur in the head and neck veins; only 1.5% of patients with thrombosis develop internal jugular vein thrombi. Malignancy, central venous catheters, and ovarian hyperstimulation syndrome have also been reported as the most common causes of internal jugular vein thrombosis, while bacteremia is unrelated [14]. According to a previous report, venous return from the astronaut’s head is reduced in space because of microgravity, causing stasis and reflux in the internal jugular veins, which may ultimately lead to internal jugular vein thrombosis [15]. Similarly, in the supine position, the influence of gravity is reduced, causing fluid shifts toward the head and increasing the internal jugular venous pressure. Nevertheless, stasis and regurgitation do not occur in the supine position alone [16]. Furthermore, aging and venous valve insufficiency may cause jugular vein regurgitation, leading to thrombus formation [17]. Virchow’s triad comprising (1) blood flow stagnation, (2) vascular endothelial damage, and (3) increased coagulation function has been associated with thrombus formation [5]. Here in addition to blood flow stagnation in the jugular vein due to host factors, such as bed rest and age, combined with increased coagulation and vascular endothelial damage caused by *S. anginosus*, the formation of a thrombus in the jugular vein based on Virchow’s triad might be observed, resulting in the invasion of *S. anginosus* into the neck tissue, and the development of Lemierre’s syndrome-like symptoms.

The utility of anticoagulation therapy in Lemierre’s syndrome is considerable controversy [18]. Although several retrospective studies have shown the efficacy of anticoagulation in Lemierre’s syndrome, no high-quality trials on the effectiveness of anticoagulants exist because Lemierre’s syndrome is a rare disorder that makes it difficult to conduct randomized controlled trials. An analysis combining 712 previously reported cases showed anticoagulation therapy reduced in-hospital new venous thromboembolism and new peripheral septic lesions with less major bleeding [19]. Meanwhile, a post hoc observational and population-based study of 82 patients revealed anticoagulation therapy did not improve the above two outcomes despite no increase in the adverse events [20]. In retrospective analyses, the confounding factors for the indication of anticoagulation therapy, such as disease severity, physician preference, facility policy, etc., impede more plausible conclusions. Concerning the possibility of septic embolization from a primary thrombus and the risk of thrombocytopenia and bleeding like our patient, it is essential to determine the indication of anticoagulation individually.

Additional significant aspect of this case was the inadequate disease activity control of rheumatoid arthritis. Rheumatoid vasculitis affects patients with rheumatoid arthritis who experience long disease duration and poor control, affecting the skin, central and peripheral nerves, eyes, lungs, heart, kidney, and gastrointestinal tract [21]. Although skin ulcers are more common on the lower legs, rheumatoid nodules may occur and ulcerate on the elbows, occiput, and sacral regions, prone to pressure irritation [22]. Moreover, in this case, the sacral lesion occurred when the patient maintained a relatively high level of daily living activity. Therefore, it is reasonable to consider the lesion as an ulcerated rheumatoid nodule rather than a pressure ulcer secondary to bedridden. Notably, after intensified treatment with increased doses of betamethasone, both the sacral and left lower leg lesions tended to shrink, consistent with ulceration due to rheumatoid vasculitis. Hence, a possibility exists that the primary infection site was derived from the inadequate treatment of rheumatoid arthritis. If the disease activity of rheumatoid arthritis had been adequately controlled, these ulcerative lesions would not have occurred, and Lemierre’s syndrome would not have developed. Therefore, it is crucial to control the disease activity of rheumatoid arthritis appropriately and prevent the ulcer lesions from worsening to avoid recurrence in these patients.

In summary, Lemierre’s syndrome is a poor prognosis disease with a high risk of inherent thromboembolism, abscess formation, and even a certain number of deaths in the present era. Additionally, atypical cases are considered challenging to diagnose due to the rare disease variant nature and require prompt and proper multidisciplinary treatment, combining antimicrobial and potentially anticoagulat and surgical therapies. Therefore, a thorough understanding of the pathophysiology of the disease should improve interventions and speed-up treatment courses.

## Data Availability

Data sharing is not applicable for this article since no datasets were generated or analyzed during the current study.

## Abbreviations

CECT: Contrast-enhanced computed tomography
CLDM: Clindamycin
CRP: C-reactive protein
MEPM: Meropenem
MRSA: Methicillin-resistant *Staphylococcus aureus*
VCM: Vancomycin
